# Ligation of the Primitive Iliac Artery

**Published:** 1852-09

**Authors:** 


*Ligation of the Primitive Iliac Artery.*—In the *New Orleans Medical and Surgical Journal* for May, is reported a case of an Irishman, aged twenty-five years, who was admitted into the Charity Hospital for aneurism of the left femoral and external iliac arteries. On the 27th of March, Dr. A. J. Wedderburn ligated the primitive iliac. During the operation "severe hæmorrhage took place;" and it was found that the aneurism extended to within one inch and a half of the bifurcation of the primitive iliac artery. No chloroform was administered, and the patient complained much during the operation. On the 29th, evidence of gangrene below the knee existed. On afternoon, the 31st, the patient died—gangrene having extended as far as the hip. Post-mortem examination revealed thickening and degeneration of the tissues about the seat of the ligature, together with enlargement of the lymphatics. Dr. Wedderburn is disposed to question the success of this operation in any case; and he doubts if a cure has ever followed this questionable operation.—*New York Journal of Medicine.*

